# Use of Intravenous Paracetamol Preoperatively Favors Lower Risk of Delirium and Functional Recovery in Elderly Patients with Hip Fracture: A Propensity Score-Matched Analysis

**DOI:** 10.1155/2022/1582727

**Published:** 2022-04-13

**Authors:** Junfei Guo, Tao Wang, Xuehong Zheng, Yubin Long, Xin Wang, Qi Zhang, Junchuan Liu, Guolei Zhang, Junpu Zha, Zhiyong Hou, Yingze Zhang

**Affiliations:** ^1^Department of Orthopaedics Surgery, Third Hospital of Hebei Medical University, Shijiazhuang, China; ^2^Orthopaedic Institute of Hebei Province, Shijiazhuang, China; ^3^Department of Orthopaedic Surgery, Hebei General Hospital, Shijiazhuang, China; ^4^Department of Orthopaedics Surgery, Baoding First Central Hospital, Baoding, China; ^5^Department of Anesthesiology, Children's Hospital of Hebei Affiliated to Hebei Medical University, Shijiazhuang, China; ^6^NHC Key Laboratory of Intelligent Orthopeadic Equipment, Shijiazhuang, China; ^7^Chinese Academy of Engineering, Beijing, China

## Abstract

We aimed to investigate whether the use of intravenous paracetamol (IVP) preoperatively in intertrochanteric fracture (IF) patients aged 65 years or over receiving intramedullary fixation had significantly benefits on the pain score at discharge, delirium incidence, length of hospital stay (LOS), functional outcomes, and mortality. A retrospective analysis of all surgically treated patients presenting with IF was conducted at a single Level I trauma center in China between Jan. 2016 and Jan. 2020. The data concerning patients' demographics, injury-related data, surgery-related data, operation-related data, in-hospital data, and postoperative outcomes were extracted. To minimize potential confounding and selection bias, the propensity score matching (PSM) method was performed via the caliper matching method by using a 1 : 1 ratio. After PSM, McNemar's chi-square tests were used to examine the association of using IVP with outcome analyses. The Spearman correlations of IVP using, pain scores, and the factors which may influence them were also computed. After screening 2963 consecutive patients, 2166 were included finally, including 1576 in the non-IVP group and 590 in the IVP group. After PSM, 531 remained in each group. The pain scores at discharge were significantly between the two groups before and after matching (all *p* < 0.001). The differences of delirium rate and functional outcomes became significant after propensity score-based matching (*p*=0.001 and 0.033, respectively), although they were not significant before matching. No significant difference was observed in other operation-related data, LOS, and crude mortality rates at 30-day, 90-day, and 12-month before and after PSM. In conclusion, this study highlights the need for preoperative IVP use to optimize pain control, postoperative functional recovery, and minimize pain-related comorbidities such as delirium in elderly patients with hip fracture.

## 1. Introduction

As the population ages and the incidence of hip fracture rises, more hip fracture patients will receive operations and effective perioperative management. Worldwide, over 1 million hip fractures occur annually, particularly increased in the developing countries. It has been reported that the number of older adults in China, i.e., people over 60 years, has reached 249 million, accounting for nearly one-fifth of the total population by 2018, with numbers projected to reach close to 450 million, more than thirty percent of the global population by 2050 [1, 2]. Nowadays, hip fractures, especially intertrochanteric fractures, which are the most common cause of orthopedic wards admission, represent a major public health concern in older adults due to multiple concurrent comorbidities and subsequent difficulty in achieving good outcomes, leading to a heavy socioeconomic pressure on society [3]. Despite substantial progress in this frail patients management over the past few decades, 1-year mortality remains high, ranging from 7% to 10% in 30 days and 12% to 35% in the first year [4, 5], and even with treatment, up to 10% of patients die postoperatively in hospital [6].

According to the literature [7, 8], effective pain management has been shown to be associated with significantly improved outcomes. Increasing evidence suggests that better pain control enables patients to start the rehabilitation process earlier and has shorter length of hospital stay (LOS), thus reducing total costs during the in-hospital period and mortality [9, 10]. Poor pain control, instead, predisposes hip fracture patients to delirium and disability, which impairs their ability to perform activities of daily living (ADL), and increases 1-year mortality and morbidity [3, 11]. In addition, previous researches [12, 13] have indicated that emotional problems such as depression and anxiety are also related to the intensity of acute pain after kinds of surgery. Therefore, pain management plays an important role in perioperative patient care since patient safety and comfort after surgery are of utmost importance when evaluating surgery procedures [14].

At the present stage, the existing evidence by the National Institute of Clinical Excellence guidelines suggests that the use of intravenous paracetamol (IVP) might be compared favourably to morphine and nerve block for analgesia as well as reaching a higher peak plasma concentration than its oral equivalent [15–17]. Currently, the most widely used analgesics are nonsteroidal anti-inflammatory drugs (NSAIDs) and opioids, or peripheral nerve blocking. However, patients who use morphine for patient-controlled analgesia experience many side effects related to opioids such as pruritus, tolerance, physical dependence, reward behavior, as well as contribute to serious and potentially permanent nerve damage [18–21]. However, relevant research regarding the use of IVP preoperatively in hip fracture patients is still relatively scarce. Furthermore, whether patients received IVP use have significantly lower delirium incidence, shorter LOS, higher survival rates, and particularly better functional outcomes are relatively lacking. Therefore, the aim of the present paper is to evaluate the preoperative use of IVP in elderly patients with intertrochanteric fractures (IF) and treated by intramedullary fixation on delirium incidence, LOS, and functional outcomes as well as mortality.

## 2. Materials and Methods

### 2.1. Study Design, Setting, and Population

A retrospective analysis of all IF patients undergoing intramedullary fixation by proximal femoral nail antirotation (PFNA) was conducted at a single Level I trauma center in China between Jan. 2016 and Jan. 2020. Patients who were 65 years or older, with an admission delay from initial injury <48 h, and received a minimum of one-year follow-up were included and screened. Exclusion criteria were open or pathological fractures, additional fractures of the IF and multiple injuries, patients who had inability to communicate, with mental illness, or refused surgery, and who were treated conservatively due to severe comorbidities, were excluded. The patients were divided into IVP or non-IVP groups according to whether they received IVP preoperatively. All investigations were conducted in conformity with the ethical principles of research. The study was overseen and approved by the institutional internal review board of the participating institution in compliance with the Declaration of Helsinki, and consent was waived as this is an observational study without an intervention. All collected patient data were anonymously recorded to protect patient confidentiality.

### 2.2. Perioperative Treatment and Surgical Procedure

Our hospital has specialized geriatric orthopedics wards. The patients in wards are assessed once daily by a multidisciplinary team including at least two orthopedists, one internal medicine consultant who is responsible for patients' perioperative management, together with an attending anesthesiologist, and nurses. Strategy in using IVP was according to the current guidelines that is given at a dose of 2000–4000 mg daily in 2–4 divided doses, which has been demonstrated had no relevant side effects on kidney and gastric function [16, 22].

Preoperative X-rays (anteroposterior and lateral view) and a Siemens 128-layer dual-source spiral CT scan (Siemens Medical System, Germany) of the injured leg were taken. Fractures were classified as stable (A1.1–A2.1) or unstable (A2.2–A3.3) according to the Orthopaedic Trauma Association classification system. The patients surgically treated by PFNA that were all following international treatment guidelines. The surgical operation was carried out under general anesthesia or region anesthesia. The position of internal fixation was checked and the wound sutured layer by layer. After the operation, early partial to full weight bearing was encouraged. The patients were followed regularly by an outpatient review or telephone interview with patients or their family members.

### 2.3. Data Collection

Data were retrospectively collected from our institution's electronic medical record. The data collection consisted of patients' demographics, including gender, age, body mass index (BMI), residence (rural or urban), and smoking or drinking history; injury-related data consisted of fracture type and time from initial injury to surgery; surgery-related data including general health status based on the American Society of Anesthesiologists (ASA) grade (ASA physical status are classified as I to VI) and modified Elixhauser comorbidity method (mECM); and in-hospital data including the Hb level at admission, whether received blood transfusion, the commonly used visual analog scores (VAS) and numerical rating scores (NRS) at admission [23, 24], Geriatric Depression Scale (GDS), functional independence measure (FIM), and anxiety or not. Outcome analyses consisted of operation-related data including anesthesia methods (general or regional), duration of operation, intraoperative blood loss; and in-hospital outcomes including VAS and NRS at discharge, LOS, and postoperative delirium or not. The participants' survival status and date of death were collected during the follow-up. Beginning of follow-up was defined as enrollment in the cohort, and end point event was defined as all reasons of death or at a most recent follow-up visit, whichever was earlier. Then, 30-day, 90-day, and 12-month mortality and functional outcomes (including independent walking, use of walking aids, wheelchair, bedridden status, and death) were also recorded.

### 2.4. Definitions

Patients' age was classified as 65–69, 70–79, 80–89, 90–99, and over 100 years old, while BMI was grouped as normal with BMI <24 kg/m^2^, overweight with 24 ≤ BMI < 28 kg/m^2^, and obesity with BMI ≥28 kg/m^2^. From electronic medical records, the mECM was used to assess patients' comorbidities at admission and further stratified into groups <0, 0, 1–5, 6–13, and ≥14 in this study cohort. Additionally, ASA grade is a commonly used predictor of mortality in orthopedic surgery. Thus, to ensure transparency, the authors have included both variables as we have done in our previously published studies [25, 26]. The 15-item GDS and FIM were used to determine the depression symptoms and the generic ability to perform ADL, respectively [27, 28]. Breakpoints of 8 g/dL, 10 g/dL, and 12 g/dL were used to classify the Hb level at admission.

### 2.5. Statistical Analysis

Continuous variables were evaluated for normality by applying the Shapiro–Wilk test. Numerical variables satisfying normality were compared using the Student *t* test to obtain group mean differences, and data are presented as mean ± standard deviation (SD). Median and interquartile range (IQR) were reported as data were non-normally distributed and done with the Mann–Whitney *U* test. Categorical variables are shown as proportions, and the differences were analyzed using the chi-square or Fisher's exact test. To reduce selection bias and potential confounding factors, propensity score matching (PSM) was adopted for the adjustment of baseline clinical by using a 1 : 1 ratio and via the caliper matching of 0.20. After PSM, paired *t* tests and paired chi-square tests were used for continuous variables and categorical variables, respectively. Finally, the Spearman correlations of IVP using, VAS, NRS, and the factors which may influence them were also computed, respectively. All data analyses were performed using IBM SPSS Statistics for Windows, version 26.0 (IBM, Armonk, NY, USA). The level of significance was set at *p* < 0.05.

## 3. Results

**Figure 1:**
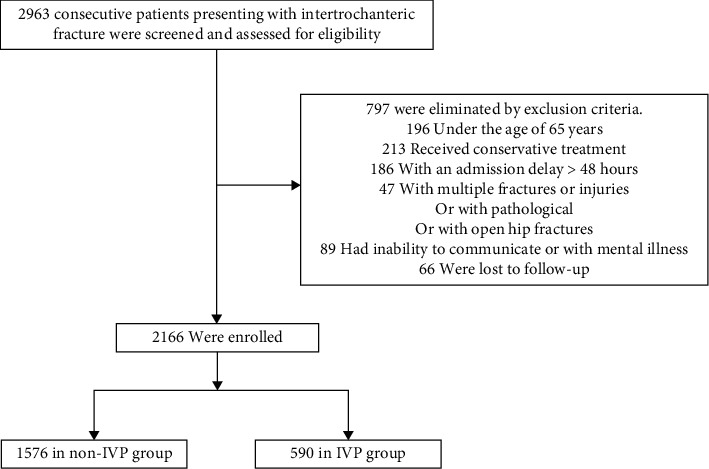
Flow diagram of included patients.

From Jan. 2016 to Jan. 2020, a total of 2963 consecutive patients presenting with IF were retrospectively reviewed and assessed for eligibility. A total of 797 patients were eliminated by the exclusion criteria. Among these patients, 196 were under 65 years old; 213 received conservative treatment; 186 had an admission delay of greater than or equal to 48 h; 47 had open hip fractures, pathological fractures, and multiple injuries; 89 had inability to communicate or with mental illness; and 66 were lost to follow-up. Finally, 2166 patients (including 1576 in the non-IVP group and 590 in the IVP group) who met the inclusion and exclusion criteria were enrolled (Figure 1).

Comparison of general data of patients between two groups is presented in Table 1. More than sixty percent of the participants were female, and the mean age was 79 years old in both groups. There were significant differences between the IVP group and the non-IVP group regarding gender, residence, smoking history, fracture type, VAS and NRS at admission, GDS, FIM, and anxiety incidence. There were 531 matched pairs after propensity score matching, and the two groups had similar baseline demographic and disease characteristics (*p* > 0.05) (Table 1).

Prematching and postmatching results, including operation-related data, VAS and NRS at discharge, LOS, delirium incidence, functional outcomes, and mortalities, are presented in Table 2. The statistical distribution showed that intraoperative blood loss was significantly different between the two groups before PSM; however, the difference was not significant after PSM. Although the differences in the characteristics of VAS and NRS at discharge were significantly reduced between the two groups, after matching, the characteristics of each covariate still differed. Notably, the differences of delirium rate and functional outcomes became significant after propensity score-based matching (*p*=0.001 and 0.033, respectively); however, before matching, the differences were not significant. No significant difference was observed in other operation-related data, LOS, and crude mortality rates at 30-day, 90-day, and 12-month before and after PSM.

To examine if IVP use, VAS, and NRS were correlated with other variables, correlation analyses were performed. By using the Spearman method, although our results showed several variables were significantly associated, pain (VAS and NRS) at admission was the only variable with the weak correlation to IVP use, while smoking and drinking histories were correlated with pain experience and severity (Tables 3, 4). The overall mortality rates of patients in the non-IVP group and the IVP-group were 20.1% and 20.5%, respectively, at the end of this study.

## 4. Discussion

It has been reported that approximately two-thirds of patients had moderate-to-severe pain before surgery [10, 29, 30]. However, to the best of our knowledge, inconsistent and inadequate pain control is indeed due to several reasons including fearing side effects, poor treatment compliance by patients, poor consistency in prescribing medications by clinicians, and underappreciated issue in patients with cognitive impairment, which is cited as a barrier to effective pain assessment [9]. In a prospective study, Oberkircher et al. found that although all patients having significant pain before arrival to the hospital, more than seventy percent of patients received no analgesia [10].

Despite oral paracetamol with various doses has been recommended routinely given as the first step of the WHO analgesic ladder [30], its bioavailability when given orally can be reduced by hepatic first pass metabolism. Instead, IVP has been proven to be a reliable and effective analgesic in managing both preoperative and postoperative pain for orthopedic patient care [31–33]. Moreover, published literatures [17, 34] have demonstrated that IVP reached a higher peak plasma concentration, with a superior opioid-sparing effect, and significantly reduced morphine requirements with no adverse effects or compromise in pain management than its oral equivalent.

This study set out to evaluate the preoperative use of IVP in elderly patients with IF and treated with intramedullary fixation, focusing on the impact of delirium incidence, LOS, functional outcomes, and mortality. Based on the results analyses performed in this study involving 2166 patients, we observed 590 patients (27.2%) received IVP treatment before surgery, and in male patients, urban living, smoking history, unstable fracture type, higher VAS, NRS, GDS at admission, anxiety, and lower FIM were independent predictors for IVP use. The intensity of acute pain after hip fracture is likely multifactorial. Numerous studies have suggested that gender [35], age [36], smoking history [35, 37], unstable fracture type [36], and anxiety [13, 37] were related factors, which are consistent with our conclusions.

Several literature focused on the use of paracetamol for pain reduction in hip fracture patients, which has been already shown to be associated with reduced delirium rate [29], mortality rate and LOS [9, 16, 29], as well as improved functional outcomes [9, 16]. Few of these studies, however, presented results that adjusted for other covariables that may confound these outcomes. Thus, we applied PSM to minimize confounding biases. The differences roughly represented the effects of gender, residence, smoking history, fracture type, VAS, NRS, GDS at admission, anxiety, and FIM on anesthesia method, intraoperative blood loss, duration of operation, VAS and NRS at discharge, LOS, delirium rate, mortality rate, and functional outcomes before PSM were eliminated in the present study. After PSM and McNemar's tests, we confirmed that IF fracture patients received IVP have advantages in terms of VAS and NRS at discharge, delirium rate, and functional outcomes than the patients who did not receive IVP before surgery. Our conclusions are in line with other literatures [10, 16] that IVP seems to have the potential to reduce delirium rate and gain better functional outcomes that possibly due to early immobilization, as a result of painless.

Previous literature [3, 16] also revealed that optimizing pain management contributed to reducing LOS while poor pain control may increase 1-year mortality. However, we did not obtain similar conclusions in this Chinese population. The fact is that there are many factors affecting LOS and mortality. Previous published articles [6, 38] studied the short-term outcomes of the elderly hip fracture patients. In this study, 437 of 2166 (20.2%) total patients died at the end of the study, and the mortality rates of the non-IVP group and IVP group in 12 months were 6.9% and 6.3% before PSM, respectively. Our findings reveal that the mortality rate is lower than previous data [5, 6, 38], which can be ascribed to the participants selection that we restricted the study population to surgical patients and excluded nonsurgical patients. Similarly, there is no significant difference in mortality rates between the two groups after PSM. Surprisingly, we found that only less than 1 in 10 of patients were restricted to a wheelchair or bedridden state requiring full assistance while most patients could walk independently/with the help of walking aids before and after PSM. Ekstrom et al. [39] demonstrated that only about 55% of patients maintain their activities of daily living and approximately 34% of patients lose their previous ability to walk. According to our results, 40.6% of non-IVP patients and 40.3% of IVP patients walked independently before PSM while the percentages were 42.7% and 44.8% after PSM, respectively. In addition, a substantial percentage of individuals are able to walk with assistive devices.

Previous considerable research has assessed the factors influencing the rates of functional outcomes and mortality in hip fracture patients. Compared to these former studies, the strength of this study lies in the more recent data with a relatively large sample size. Other strengths are the sets of scoring systems we involved and the specific cohort of patients who received surgery by a single internal fixation and grouped based on whether they received IVP or not, which eliminated the effects of possible confounding variables. Finally, to the best of our knowledge, this is the first study to evaluate IVP on functional outcomes in hip fracture patients after PSM. Such quantitative analyses might increase the orthopedist's confidence in pain management for hip fracture patients and be beneficial for clinicians looking to establish probabilities for delirium and adverse functional outcomes in the future and establishing rational goals of medical care for this vulnerable population. A weakness, however, comes with the fact that it is a retrospective single-center observational study. In addition, we did not rule out other unknown factors, including perioperative laboratory values and surgeon practice, for analysis, which may potentially influence our findings. However, this is the first study including multiple relative contributions of patient demographics, injury-related, surgery-related, anesthetic, transfusion, and sets of scoring systems factors, which have not been studied together.

## 5. Conclusion

In conclusion, early identification of individuals with moderate-to-severe pain and using IVP preoperatively for pain killing is prone to reducing pain score at discharge, delirium incidence, and achieving better functional outcomes that benefited from accelerated care. This study highlights the need for preoperative IVP to optimize pain control and minimize pain-related comorbidity as well as postoperative functional recovery in elderly patients with hip fracture.

## Figures and Tables

**Table 1:** Patient characteristics at baseline comparisons before and after propensity score matching.

Variables	Prematching			Postmatching		
	Non-IVP group (*n* = 1576)	IVP group (*n* = 590)	*p* value	Non-IVP group (*n* = 531)	IVP group (*n* = 531)	*p* value
*Demographics*						
Gender, *n* (%)			0.043^*∗*^			0.565
Male	494 (31.3%)	212 (35.9%)		187 (35.2%)	196 (36.9z5)	
Female	1082 (68.7%)	378 (64.1%)		344 (64.8%)	335 (63.1%)	
Age, years	79.0 ± 7.2	79.2 ± 7.3	0.510	79.0 ± 7.4	79.3 ± 7.2	0.518
Age group, *n* (%)			0.142			0.174
65–69	172 (10.9%)	60 (10.2%)		60 (11.3%)	51 (9.6%)	
70–79	639 (40.5%)	230 (39.0%)		218 (41.1%)	211 (39.7%)	
80–89	655 (41.6%)	251 (42.5%)		215 (40.5%)	225 (42.4%)	
90–99	103 (6.5%)	49 (8.3%)		34 (6.4%)	44 (8.3%)	
≥100	7 (0.4%)	0 (0.0%)		4 (0.8%)	0 (0.0%)	
BMI (kg/m^2^), *n* (%)			0.937			0.939
Normal (BMI < 24)	1023 (64.9%)	384 (65.1%)		345 (65.0%)	347 (65.3%)	
Overweight (24 ≤ BMI < 28)	431 (27.3%)	163 (27.6%)		146 (27.5%)	147 (27.7%)	
Obesity (BMI ≥ 28)	122 (7.7%)	43 (7.3%)		40 (7.5%)	37 (7.0%)	
Residence			<0.001^*∗*^			0.413
Rural	670 (42.5%)	189 (32.0%)		199 (37.5%)	212 (39.9%)	
Urban	906 (57.5%)	401 (68.0%)		332 (62.5%)	319 (60.1%)	
Smoking history (Yes)	186 (11.8%)	90 (15.3%)	0.032^*∗*^	87 (16.4%)	78 (14.7%)	0.446
Drinking history (Yes)	373 (23.7%)	157 (26.6%)	0.156	130 (24.5%)	134 (25.2%0	0.776

*Injury-related data*						
Fracture type, *n* (%)			<0.001^*∗*^			0.422
Stable (A1.1–A2.1)	928 (58.9%)	252 (42.7%)		244 (46.0%)	231 (43.5%)	
Unstable (A2.2–A3.3)	648 (41.1%)	338 (57.3%)		287 (54.0%)	300 (56.5%)	
Time from injury to surgery, days	6.1 ± 3.1	5.9 ± 3.3	0.100	6.0 ± 3.1	6.0 ± 3.3	0.766

*Surgery-related data*						
ASA, *n* (%)			0.713			0.859
1	306 (19.4%)	110 (18.6%)		101 (19.0%)	99 (18.6%)	
2	451 (28.6%)	184 (31.2%)		152 (28.6%0	164 (30.9%)	
3	575 (36.5%)	200 (33.9%)		196 (36.9%)	181 (34.1%)	
4	206 (13.1%)	82 (13.9%)		72 (13.6%)	75 (14.1%)	
5	38 (2.4%)	14 (2.4%)		10 (1.9%0	12 (2.3%)	
mECM, *n* (%)			0.580			0.752
<0	31 (2.0%)	13 (2.2%)		12 (2.3%)	13 (2.4%)	
0	804 (51.0%)	278 (47.1%)		272 (51.2%)	250 (47.1%)	
1–5	257 (16.3%)	99 (16.8%)		86 (16.2%)	90 (16.9%)	
6–13	420 (26.6%)	174 (29.5%)		140 (26.4%)	155 (29.2%)	
≥14	64 (4.1%)	26 (4.4%)		21 (4.0%)	23 (4.3%)	

*In-hospital data*						
Hb level at admission (g/dL)			0.729			0.962
Hb ≥ 12	454 (28.8%)	179 (30.3%)		155 (29.2%)	160 (30.1%)	
12 > Hb ≥ 10	670 (42.5%)	237 (40.2%)		219 (41.2%)	215 (40.5%)	
10 > Hb ≥ 8	371 (23.5%)	146 (24.7%)		127 (23.9%)	129 (24.3%)	
Hb < 8	81 (5.1%)	28 (4.7%)		30 (5.6%)	27 (5.1%)	
Blood transfusion (Yes)	1203 (76.3%)	149 (74.7%)	0.442	413 (77.8%)	400 (75.3%)	0.346
VAS at admission	4.9 ± 1.7	6.4 ± 1.5	<0.001^*∗*^	5.9 ± 1.3	6.0 ± 1.6	0.410
NRS at admission	4.9 ± 1.7	6.4 ± 1.5	<0.001^*∗*^	5.9 ± 1.3	5.9 ± 1.6	0.934
GDS	3.5 ± 1.6	4.4 ± 1.3	<0.001^*∗*^	4.0 ± 1.9	4.2 ± 1.7	0.109
FIM	84.6 ± 10.6	83.4 ± 10.2	0.022^*∗*^	83.1 ± 10.2	83.3 ± 10.9	0.769
Anxiety (Yes)	142 (9.0%)	507 (14.1%)	0.001^*∗*^	102 (19.2%)	82 (15.4%)	0.105

Values are presented as the number (%) or mean ± SD (standard deviation). ^*∗*^*p* < 0.05, statistical significance. BMI, body mass index; ASA, American Society of Anesthesiologists; mECM, modified Elixhauser's Comorbidity Measure; VAS, visual analog scores; NRS, numerical rating scores; GDS, Geriatric Depression Scale; FIM, functional independence measure.

**Table 2:** Patient outcome analyses before and after propensity score matching.

Variables	Prematching			Postmatching		
	Non-IVP group (*n* = 1576)	IVP group (*n* = 590)	*p* value	Non-IVP group (*n* = 531)	IVP group (*n* = 531)	*p* value
Type of anesthesia, *n* (%)			0.906			0.849
General anesthesia	592 (37.6%)	220 (37.3%)		197 (37.1%)	200 (37.7%)	
Regional anesthesia	984 (62.4%)	370 (62.7%)		334 (62.9%)	331 (62.3%)	
Duration of operation, mins	99.7 ± 34.7	97.2 ± 34.6	0.130	99.8 ± 34.5	97.8 ± 35.0	0.352
Intraoperative blood loss (mL)	200 (100, 300)	200 (100, 300)	0.012^*∗*^	200 (100, 300)	200 (100, 300)	0.095
VAS at discharge	2.4 ± 1.1	1.5 ± 0.9	<0.001^*∗*^	2.3 ± 1.1	1.8 ± 0.9	<0.001^*∗*^
NRS at discharge	2.3 ± 1.1	1.4 ± 0.8	<0.001^*∗*^	2.2 ± 1.1	1.8 ± 0.9	<0.001^*∗*^
Length of hospital stay	14.7 ± 6.8	14.3 ± 6.0	0.255	14.3 ± 6.5	14.4 ± 6.1	0.815
Delirium (Yes)	128 (8.1%)	48 (8.1%)	0.992	85 (16.0%)	48 (9.0%)	0.001^*∗*^
30-day mortality	14 (0.9%)	5 (0.8%)	0.928	6 (1.1%)	5 (0.9%)	0.762
90-day mortality	25 (1.6%)	8 (1.4%)	0.697	10 (1.9%)	8 (1.5%)	0.634
12-month mortality	109 (6.9%)	37 (6.3%)	0.594	39 (7.3%)	37 (7.0%)	0.812

Functional outcomes			0.984			0.033^*∗*^
Independent walking	640 (40.6%)	238 (40.3%)		227 (42.7%)	238 (44.8%)	
Use of walking aids	488 (31.0%)	182 (30.8%)		158 (29.8%)	178 (33.5%)	
Use of wheelchair	92 (5.8%)	32 (5.4%)		34 (6.4%)	41 (7.7%)	
Bedridden	40 (2.5%)	17 (2.9%)		15 (2.8%)	7 (1.3%)	
Death	316 (20.1%)	121 (20.5%)		97 (18.3%)	67 (12.6%)	

Values are presented as the number (%) or mean ± SD (standard deviation) or median (interquartile range). ^*∗*^*p* < 0.05, statistical significance. VAS, visual analog scores; NRS, numerical rating scores.

**Table 3:** The association of IVP group with gender, age, residence, smoking or drinking history, fracture type, pain scores at admission, GDS, FIM, and anxiety.

Variables	Non-IVP group (*n* = 1576)	IVP group (*n* = 590)	Spearman's r statistic	*p* value
Gender, *n* (%)			−0.044	0.043^*∗*^
Male	494 (31.3%)	212 (35.9%)		
Female	1082 (68.7%)	378 (64.1%)		

Age, years	79.0 ± 7.2	79.2 ± 7.3	0.016	0.451
Age group, *n* (%)			0.026	0.235
65–69	172 (10.9%)	60 (10.2%)		
70–79	639 (40.5%)	230 (39.0%)		
80–89	655 (41.6%)	251 (42.5%)		
90–99	103 (6.5%)	49 (8.3%)		
≥100	7 (0.4%)	0 (0.0%)		

Residence			−0.096	<0.001^*∗*^
Rural	670 (42.5%)	189 (32.0%)		
Urban	906 (57.5%)	401 (68.0%)		

Smoking history (Yes)	186 (11.8%)	90 (15.3%)	0.049	0.022^*∗*^
Drinking history (Yes)	373 (23.7%)	157 (26.6%)	0.033	0.126

Fracture type, *n* (%)			0.147	<0.001^*∗*^
Stable (A1.1–A2.1)	928 (58.9%)	252 (42.7%)		
Unstable (A2.2–A3.3)	648 (41.1%)	338 (57.3%)		
VAS at admission	4.9 ± 1.7	6.4 ± 1.5	0.388	<0.001^*∗*^
NRS at admission	4.9 ± 1.7	6.4 ± 1.5	0.396	<0.001^*∗*^
GDS	4.4 ± 1.3	3.5 ± 1.6	−0.286	<0.001^*∗*^
FIM	84.6 ± 10.6	83.4 ± 10.2	0.055	0.010^*∗*^
Anxiety (Yes)	142 (9.0%)	507 (14.1%)	0.074	0.001^*∗*^

Values are presented as the number (%) or mean ± SD (standard deviation). ^*∗*^*p* < 0.05, statistical significance. VAS, visual analog scores; NRS, numerical rating scores; GDS, Geriatric Depression Scale; FIM, functional independence measure.

**Table 4:** The association of pain scores (VAS and NRS) with gender, age, BMI, residence, smoking or drinking history, fracture type, ASA, mECM, Hb level at admission, GDS, FIM, and anxiety.

Variables	VAS at admission	*p* value	NRS at admission	*p* value
Gender	−0.011	0.595	−0.004	0.836
Age	0.002	0.917	0.003	0.874
Age group	0.002	0.915	0.004	0.863
BMI	0.003	0.895	−0.002	0.924
Residence	−0.095	<0.001^*∗*^	−0.087	<0.001^*∗*^
Smoking history (Yes)	0.209	<0.001^*∗*^	0.201	<0.001^*∗*^
Drinking history (Yes)	0.262	<0.001^*∗*^	0.292	<0.001^*∗*^
Fracture type	0.015	0.474	0.015	0.497
ASA	0.008	0.726	0.010	0.638
mECM	−0.007	0.750	−0.004	0.863
Hb level at admission	0.025	0.242	0.024	0.263
GDS	−0.157	<0.001^*∗*^	−0.166	<0.001^*∗*^
FIM	−0.088	<0.001^*∗*^	−0.099	<0.001^*∗*^
Anxiety	0.010	0.644	0.020	0.348

^*∗*^ *p* < 0.05, statistical significance. VAS, visual analog scores; NRS, numerical rating scores; BMI, body mass index; ASA, American Society of Anesthesiologists; mECM, modified Elixhauser's Comorbidity Measure; GDS, Geriatric Depression Scale; FIM, functional independence measure.

## Data Availability

The data and code used to support the findings of this study are available from the corresponding author upon request.

## Abbreviations

LOS: Length of hospital stay
ADL: Activities of daily living
IVP: Intravenous paracetamol
IF: Intertrochanteric fracture
PFNA: Proximal femoral nail antirotation
BMI: Body mass index
ASA: American Society of Anesthesiologists
mECM: Modified Elixhauser comorbidity method
VAS: Visual analog scores
NRS: Numerical rating scores
GDS: Geriatric Depression Scale
FIM: Functional independence measure
PSM: Propensity score matching.
